# Shoulder arthralgia: case study of the Occupational Medicine clinic of Universidade Estadual de Campinas (Unicamp)

**DOI:** 10.47626/1679-4435-2022-789

**Published:** 2023-02-13

**Authors:** William Prado, Valmir Azevedo, Sérgio Roberto de Lucca, Marcia Bandini

**Affiliations:** 1 Departamento de Saúde Coletiva, Área de Saúde do Trabalhador, Faculdade de Ciências Médicas, Universidade Estadual de Campinas, Campinas, SP, Brazil

**Keywords:** rotator cuff injuries, occupational health, ergonomics, diagnosis, rehabilitation, lesões do manguito rotador, saúde do trabalhador, ergonomia, diagnóstico, reabilitação

## Abstract

**Introduction:**

Shoulder pain is ranked as the third most common musculoskeletal complaint in clinical practice. It is estimated that 65 to 70% of these occurrences are due to rotator cuff injuries. A significant number of rotator cuff syndrome cases are work related.

**Objectives:**

To evaluate the success or failure of therapeutic and administrative procedures for workers treated at an occupational medicine outpatient clinic.

**Methods:**

This study analyzed the medical reports of 142 workers treated for shoulder pain between January 2015 and December 2019. To homogenize the information, medical record review was necessary in some cases.

**Results:**

Rotator cuff syndrome was diagnosed in 84% of the cases after imaging exams. Conservative treatment was recommended for 88% of these patients and 58% required subsequent surgical treatment. Regarding rehabilitation, 51% of the patients were able to return to work and 49% returned to the same job function.

**Conclusions:**

Diagnosing rotator cuff syndrome requires clinical and occupational history assessment, as well as imaging examinations, and the sensitivity and specificity of ultrasound were similar to magnetic resonance imaging. Removal from work and its risks must be an integral part of treatment. Upon returning to work, the rehabilitation and reintegration process should involve activities that will not worsen the injury.

## Abstract (pt)

**Introdução:**

De acordo com a literatura médica, as dores nos ombros são a terceira queixa musculoesquelética mais comum observada na prática clínica. Calcula-se que 65 a 70% dessas ocorrências sejam causadas por lesões do manguito rotador. Um número significativo de casos da síndrome do manguito rotador está relacionado ao trabalho.

**Objetivos:**

Avaliar os diagnósticos dessa síndrome e os fatores de sucesso e insucesso nas condutas terapêuticas e administrativas de trabalhadores atendidos em um ambulatório de Medicina do Trabalho.

**Métodos:**

Este estudo analisou 142 relatórios médicos de trabalhadores com queixa de dores nos ombros atendidos no ambulatório de Medicina do Trabalho, de janeiro de 2015 a dezembro de 2019. Para homogeneizar as informações, em alguns casos, foi necessária a revisão do prontuário médico.

**Resultados:**

Após a realização de exames de imagem, 84% dos casos tiveram diagnóstico confirmado de síndrome do manguito rotador. Em 88% dos casos, foi indicado tratamento conservador, e parte destes (58%) evoluiu para tratamento cirúrgico. No processo de reabilitação, 51% dos pacientes foram reinseridos em atividade compatíveis e/ou readaptados ao trabalho e 49% não tiveram mudança de função.

**Conclusões:**

Além da história clínica e ocupacional, o exame de ultrassonografia teve sensibilidade e especificidade semelhantes à ressonância magnética para o diagnóstico de síndrome do manguito rotador. O afastamento do trabalho e dos fatores de risco ergonômicos devem ser parte integrante do tratamento da síndrome do manguito rotador. No retorno ao trabalho, o processo de reinserção e reabilitação precisa considerar a atividade compatível para não haver agravamentos das lesões.

## INTRODUCTION

Greater competitiveness and productivity is a global trend that has led to a faster work pace and fewer/shorter breaks during shifts. In this context, musculoskeletal overload increases the risk of repetitive strain injuries and work-related musculoskeletal disorders (RSI/WRMD).^1^

An increased incidence of different clinical forms of RSI/WMSD has been observed among workers in several countries, negatively impacting health and productivity. In the United States, Scandinavia, and Japan, RSI/WMSD represents approximately 30% of all registered occupational diseases and is the leading cause of inability to participate in work activities.^2^

In Brazil, RSI/WRMD was the second leading cause of accident benefits claims between 2011 and 2013^2^. Between 2012 and 2018, shoulder injuries were the leading cause of accident-related sick leaves and corresponded to 17% (98,437) of all WMSD sick leaves.^3,4^

The medical literature ranks shoulder pain as the third most common musculoskeletal complaint observed in clinical practice.^5^ It is estimated that 65 to 70% of these occurrences are caused by rotator cuff injuries.^6^

Studies show that working conditions and inadequate ergonomic positions are the main triggers of shoulder pain and limitations, especially activities that demand repetitive movements, movements above the shoulder line, static and prolonged contraction, inadequate postures, and applying force.^7^ The cumulative effect of musculoskeletal overload due to work activities increases the risk of shoulder injuries and can be approximately 4 times greater when workers are exposed to a combination of three physical factors (eg, force, awkward posture, and overload) for at least 3 months.^7^

In the United States, a higher prevalence of clinically defined rotator cuff syndrome was observed among workers who performed work activities that included a combination of physical overload factors (eg, long periods of shoulder flexion and vigorous exertion) than those exposed to a single factor.^7^ A cross-sectional study of French workers found that shoulder pain was directly associated with biomechanical exposure, while factors related to work organization and psychosocial factors indirectly affected the risk of chronic shoulder pain.^8^

Rotator cuff syndrome (RCS) is the most common condition of the shoulder girdle, which consists of the supraspinatus, infraspinatus, subscapularis, and teres minor muscles. Impairment of these muscles is usually related to work activities or a traumatic event.^9^ In this syndrome, shoulder pain can cause varying degrees of functional impairment depending on the affected muscle(s). It is the main complaint of 15-25% of patients who seek orthopedic treatment and physical therapy.^1^

Occupational history, physical examination, and specific procedures to evaluate the affected muscles are fundamental for the diagnostic hypothesis,^9-11^ which can be confirmed through imaging tests and results in an indication for conservative or surgical treatment.^12^ These tests should involve the appropriate sensitivity and specificity for the investigated structures. Using the proper technique, ultrasound and magnetic resonance imaging (MRI) have similar sensitivities for evaluating the rotator cuff and surrounding soft tissues.^13^

According to the literature, conservative treatment without surgical intervention can be successful, provided sufficient rest, functional gain, muscle strengthening, and a return to compatible activity.^14^ Treatment for rotator cuff injuries is typically conservative, including physical therapy focused on strengthening the scapular and central muscles.^15^ After treatment, adequate reintegration to the workplace is necessary. In this process, the actions necessary for reintegration and/or rehabilitation are challenging for medical assistants and occupational physicians, because they must consider the triggering factors, which often remain uncontrolled.^16^

If returning to the original work activity is impossible, reassignment depends on the variety of jobs offered by the organization, the appropriate reception, and the changes necessary to expand the worker’s reintegration possibilities, which involve agreement between the worker, the company, other workers, and government agencies.^17^

This study aimed to evaluate the diagnosis and the success or failure of therapeutic and administrative procedures for workers with shoulder pain treated at a WRMD outpatient clinic.

## METHODS

The occupational medicine outpatient clinic of Universidade Estadual de Campinas (Campinas state university hospital) assists workers in the metropolitan region of Campinas, SP, Brazil. At the clinic, cases are handled by interns and/or residents under faculty supervision. Once the diagnostic hypothesis has been confirmed, patients are issued a detailed medical report that includes their clinical and occupational history, a description of the physical examination and musculoskeletal workup, the results of complementary examinations, especially imaging examinations, the diagnosis, and recommendations for clinical, administrative, and occupational procedures.

This case study used secondary data from 142 medical reports of shoulder pain cases treated at the clinic between January 2015 and December 2019. The extracted data were organized in an Excel spreadsheet using the following variables: year of admission, main complaint, pain start date, reported ergonomic risk factors, physical examination data, imaging test results, diagnosis, treatment, sick leave, change in job description, benefits, occupation according to Classificação Brasileira de Ocupações (Brazilian classification of occupations),^18^ and the company’s field of activity according to the Classificação Nacional de Atividades Econômicas (Brazilian classification of economic activities - CNAE).^19^ When necessary and to homogenize the information contained in the reports, the patient’s medical charts were consulted. This study was approved by Universidade Estadual de Campinas Research Ethics Committee (number 36407720.7.0000.5404).

## RESULTS

According to CNAE, approximately 90% of the sample work or worked in metallurgy/transformation industry. Regarding the location of the shoulder pain, 63% was bilateral, 27% was in the right shoulder only, and 10% was in the left shoulder only.

During the occupational anamnesis, the main reported ergonomic risk factors were repetitive movement (24%), application of force plus repetitive movement (20%), application of force plus inadequate posture (14%), repetitive movement plus inadequate posture (11%), application of force (10%), and other combinations of ergonomic risk factors (21%).

According to the workers, the pain began after a variable period of work activity: more than 3 years (62%), 1-3 years (34%), and less than 1 year (4%). A total of 229 imaging exams were performed for diagnostic confirmation, of which 139 MRI and 81 ultrasound results were abnormal. A total of 43% of the patients were diagnosed with bilateral RCS, 27% with RCS in the right shoulder, and 14% with RCS in the left shoulder.

Most of the workers did not immediately seek treatment due to fear of being fired. Conservative treatment was proposed in 88% of cases, while 12% were directly referred for surgery. The duration of conservative treatment also varied: more than 2 years (46%), from 7 months to 2 years (42%), and less than 7 months (12%). Conservative treatment was ineffective for 58% of the workers, who subsequently underwent arthroscopic surgery. Although the procedure type was not clearly identified due to being performed outside our hospital and to being rarely described in assistant physician reports, 52% of the surgeries were on the right shoulder, 29% were on the left shoulder, and 19% were on both. Of those who underwent surgery, 26% were reoperated.

Irrespective of treatment type, 84% of the workers were on leave for more than 15 days. Of the benefits granted, 51% were type B91 (accident-illness), 22% were type B31 (social security), and another 23% were legally converted from B31 to B91, while 4% were type B94 (accident sequelae).

After sick leave, 49% of the workers returned to the same work activities, which involved the same ergonomic risk factors. Of those who changed jobs, 31% were transferred to a compatible activity at the employer’s initiative and 20% were formally transferred through the intervention of Instituto Nacional de Seguro Social (National Social Security Institute - INSS).

## DISCUSSION

The approach to RSI/WMSD at our reference center for outpatient occupational medicine involves consideration of clinical, epidemiological, social security, and labor factors, which demonstrates its complexity and the number of agents involved. When diagnosing RSI/WMSD, occupational physicians must provide guidance about treatment, recovery, rehabilitation, and reinstatement in work. Thus, the causal link must be investigated and, when necessary, patients must be removed from work activities to avoid aggravating their condition.

An adequate diagnosis requires comprehensive and detailed clinical and occupational anamneses, investigation of ergonomic factors related to work activity, general and specific physical examinations involving appropriate tests, as well as complementary examinations to confirm the diagnosis and determine the best treatment type.

During anamnesis, the patients reported exposure to several ergonomic risk factors, notably repetitive movement associated or not with the application of force or inadequate posture, which reinforces the importance of cumulative trauma in the pathophysiology of musculoskeletal diseases. For some reports, the risk assessment could be carried out *in loco*, establishing a causal link; however, when access to the company’s facilities was not allowed, the workers’ report of clinical-epidemiological criteria was essential for studying the association between work conditions and the disorder. In 74% of the cases in which a sick leave was granted, the INSS’ Nexo Técnico Epidemiológico Previdenciário (technical nexus of social security epidemiology) was applied. This statistical tool is used to associate diseases/accident types with professional activities, helping eliminate underreporting.

When investigating RCS, MRI and ultrasound, in addition to confirming the diagnosis, can help determine whether conservative or surgical treatment is necessary.^20^ Although MRI is considered the gold standard for investigating shoulder pain,^13^ its expense can be prohibitive. The accuracy of ultrasound has improved, yielding similar results to MRI for diagnosing full- and partial-thickness rotator cuff tears at a much lower cost.^21^ In a retrospective study of 61 patients with shoulder pain who underwent preoperative ultrasound or MRI, the sensitivity and specificity of ultrasound were 87% and 63%, respectively, compared to 95% and 72% for MRI, respectively.^20^ In another study on rotator cuff tear detection, the sensitivity and accuracy of ultrasound were 92.2 and 89%, compared to 96.4 and 90% for MRI, respectively.^22^ Therefore, given its greater availability and lower cost, ultrasound seems advantageous in RCS diagnosis.

In our study, 84% of the workers were diagnosed with RCS. This corroborates the results of Doiron-Cadrin et al.,^23^ confirming that rotator cuff disorders are the most frequent group of pathologies to affect the shoulder, representing 50-85% of shoulder disorders treated by health care professionals. It has been reported that shoulder injuries are the main cause of WMSD in Brazil, especially in the metallurgical sector,^24^ which corroborates our results.

Regardless of treatment type, it is of fundamental importance to remove the patient from work activities and, thus, the risk factors that caused the injury. In our sample, 16% of the patients were still at work. Such a situation, often determined or agreed to by the occupational physician and/or orthopedist, can both impede worker recovery and worsen the injury. Among our cases, inefficient technical procedures and too few physical therapy sessions or discontinuation thereof contributed to chronic shoulder pain.

Surgery is indicated when patients do not recover after 6 months of conservative treatment, when partial rupture reaches more than 50% of the tendon thickness, or when the rupture is complete.^14^ In this study, 58% of the workers who received conservative treatment subsequently required surgical intervention. Of these, 53% went on sick leave during conservative treatment and 47% did not.

In a review by Garving et al.,^25^ 2 years of conservative treatment produced satisfactory results in 60% of the cases.^25^ Depending on the severity of the injury, the duration of conservative treatment can vary from 3 to 6 months.^23^ According to other studies in this review, the evidence was insufficient to determine whether conservative or surgical treatment was more appropriate.^25^ A meta-analysis comparing surgical and conservative treatment at 2 years of follow-up could not conclude which treatment type is better. The authors concluded that randomized clinical trials should be conducted due to the heterogeneity of the current findings.^26^ After a six-month postsurgical leave, patients can return to their usual work activities.^27^

Regarding work leaves during RSI/WRMD treatment, two professionals play a prominent role. The first is the occupational physician, who makes the diagnosis, investigates the causal link, orients the employer regarding corrective and preventive actions, and notifies the Disease Information System. The second is the INSS expert physician, whose primary responsibility is to assess the relevance of granting social security benefits to the worker. Both may face difficulties and restrictions in performing these tasks. Occupational physicians must fulfill their technical and ethical duties, including removing the worker from risk and notifying the work-related injury system. The expert physician must evaluate the insured parties, the reports issued, the work accident documentation, if any, the involved epidemiological criteria and whether they should be input into the system, and oversee the granting of benefits. Moreover, the expert physician must determine whether the worker should be referred for rehabilitation.

The fact that 16% of the workers in our sample were not put on sick leave during treatment is worrying and provides an opportunity for ethical reflection on the conduct of the attending physician and/or the company, since continuing risky work activity can aggravate the injury. Another worrisome aspect was that 49% of the workers returned to the same work activity, meaning that without effective ergonomic interventions their clinical condition could worsen.

In addition to the clinical and objective aspects of treatment management, workers affected by RSI/WRMD also experience emotional exhaustion since they must fight for recognition of their disorder. Those on INSS sick leave must still undergo numerous consultations and reviews to prove their inability to work in a context of conflicting experts and reports, especially regarding whether or not they are fit to return to work.^28^

Studies show that returning to work is a challenge for the worker, the employer, and the INSS. The reintegration process can be a chance to restart productive life with satisfaction, pleasure, and health or, on the contrary, could give rise to new disorders, worsening symptoms, and chronic disease, which compromise not only work relationships, but self-esteem and future prospects for healthy work.^29^

Proper management of WMSD cases is not easy since the patient cannot return to the work activities that triggered the injury. In the reintegration process, the occupational physician must consider changes in the workplace or in recommending another activity compatible with the worker’s clinical condition to avoid aggravation and further leaves. This process should also occur progressively and gradually for better adaptation.

Returning to the disorder’s place of origin can be an arduous experience for workers, since they cannot achieve their former level of performance. This gives rise to feelings of helplessness, frustration and fear, since, in addition to disrespect for their new clinical condition, workers must also deal with the judgment of colleagues who think that a worker who performs at a lower level should receive a lower salary. Injured workers often feel pressure from their boss, who doubts their limitations due to ignorance about the gradual nature of the reintegration process, attributing lower production to laziness. Not infrequently, such workers are victims of harassment. If the work and risk conditions are not changed, it is likely that the worker’s condition could recur or worsen.

Organizations must develop effective reintegration programs, preferably with a multidisciplinary team that can assess the worker’s limitations and possibilities, establishing gradual goals for each case. The INSS professional rehabilitation program should include all eligible workers, thus fulfilling their constitutional rights.^17,29^

In our sample, only 20% of the workers were formally rehabilitated by the INSS. This may be due in part to the alleged difficulties companies have in making compatible positions available. However, this could also be an INSS management problem, since expert physicians allege difficulties in referring the insured to the professional rehabilitation program, which itself has suffered recent cutbacks.^17,30^ In such a scenario, transferring this responsibility to companies weakens the reintegration process, since they often cannot provide compatible positions whose conditions will avoid aggravating the injury. The inferior positions they do offer are often a discredit to the worker’s intellectual capacity. Thus, INSS rehabilitation processes must guarantee the worker’s right to stability, and readaptation procedures must consider the particularities of each case.

The disease, sick leave, and reintegration process is directly related to the job itself and the relationships that flow from it. Even when the work activity itself is not the main factor in the illness (and, thus, the sick leave), the organization and its agents have a decisive role in the reintegration process.^31^

This study is limited due to convenience sampling of workers treated at a single clinic of one university hospital. Most of the sample had more severe WMSD and were generally discouraged by the stigma resulting from their chronic condition.

## CONCLUSIONS

We found that RCS was the main work-related disorder among workers treated at a reference hospital for WRMD in the Campinas region. About 90% of the sample consisted of metallurgical workers, all of whom reported exposure to ergonomic risk factors for shoulder injuries. Conservative treatment was indicated for 88% of the cases, while 12% underwent surgery as a first option. Of those who underwent conservative treatment, 58% subsequently underwent a surgical procedure. Of the total sample, 16% did not go on sick leave during treatment. Of those who did, 51% received social security benefits (B91). Even with the causal link recognized, 49% of the workers returned to the same work activity after rehabilitation.

We point out that removal from work and risk should be an integral part of treatment. The rehabilitation and reintegration process must consider compatible activities to avoid aggravating the injury.

This study can help improve care at our clinic and standardize the preparation of medical reports. It can also lead occupational physicians from public and private companies and INSS expert physicians to reflect on their responsibilities in this process. It is essential that the technical, ethical, and legal responsibilities are met so that workers receive better care and their condition is not aggravated, which also impacts mental health.
